# Awake video assisted thoracic surgery series report

**DOI:** 10.1186/1749-8090-8-S1-O243

**Published:** 2013-09-11

**Authors:** A Klijian, N Andonian

**Affiliations:** 1 Sharp & Scripps Healthcare, San Diego, CA, USA

## Background

Traditionally, video-assisted thoracic surgery (VATS) is performed under general anesthesia with selective ventilation and endotracheal intubation. Although some sparse data exists on VATS under local anesthesia, most series reserve this technique for pleural-based surgery. This technique includes more complex procedures, with results that surpass traditional open thoracotomies.

## Methods

This case study analyzed 196 patients who underwent awake thoracic surgery from June 2010 to October 2012. This single surgeon experience includes wedge resections, lobectomies, decortications, pleural biopsies, pleurodesis, bullectomies and pericardial windows.

## Results

Of the 196 cases, sixty were wedge resections, with an average length of hospital stay (ALS) of 1.5 days. ALS for the 38 decortications was 2.5 days, for the 28 pleural biopsies was 1 day. There were 36 mechanical and talc pleurodesis for recurrent effusions, 30 of them malignant, having an ALS of 1.5 days. Two pericardial windows were performed, with an ALS of 1.5 days. Twenty-two patients had lobectomies for malignancies (6 left upper lobes, 7 left lower lobes, 5 right upper lobes, 1 right middle lobe, 2 right lower lobes and 1 left lower lobe with lingulectomy), with an ALS of 1.8 days.

## Conclusions

The results of operating under local anesthesia with sedation provide comparable, if not better, post-operative results. By choosing not to subject patients to general anesthesia with an endotracheal tube and one lung ventilation, as well as providing a lung sealant (Progel®), we shortened the average hospital stay, provided quicker recovery times, better patient satisfaction and presumably cost savings. We recommend that surgeons experienced in VATS procedures attempt to operate while their patients are merely sedated. And if that emergent intubation were required, necessary equipment, personnel and patient positioning are taken into consideration before beginning the procedure.

